# Chorea and Its Treatment

**Published:** 1915-05

**Authors:** G. Wilse Robinson

**Affiliations:** Kansas City, Missouri


### Chorea and its Treatment.

BY G. WILSE ROBINSON, M. D., KANSAS CITY, MISSOURI.

Chorea belongs to that class of diseases of which is characterized for the most part by the presence of some variety of muscular spasm, the term spasm being applied to muscular contraction which is in excess of that occurring in conditions of health.

The principal diseases coming under this classification are: First, spasm of the muscle supplied by the cranial nerve; second, spasms affecting the muscles of the trunk and the limb; third, paramyo-clonus multiplex; fourth, tics or habit spasms; fifth, spasmodic torticalis; sixth, tetany; seventh, paralysis agitans; eighth, Thompson's disease; ninth, Sydenham's chorea; tenth, Huntington's chorea; eleventh, senile chorea.

There has been much speculation and investigation relative to the regional pathology of these conditions. The general conclusions drawn are, that this is not always the same; but the consensus of opinion is that it is usually extra-pyramidal, paralysis usually not being an associated symptom. The spasms constituting the convulsions of epilepsy result from irritation of the cortical cells of the cerebrum. Compression of the seventh nerve at the base of the skull may be a cause of spasm of the facial muscles, as may a meningitis involving the third nerve cause a spasmodic squint, the former spasms being due to irritation of the upper motor neurons, the two latter due to irritation of the lower motor nuerons. But the same results may obtain from a reflex irritation as well as from a direct irritation.

In the regulation of muscle tone, the cerebrum and cerebellum are antagonistic. The cerebral cortex inhibits muscle tone, while the cerebellum exerts a tonic influence upon the muscles. In order, therefore, that muscle tone may maintain its normal standard, it is necessary that the cerebellum and cerebrum functionate harmoniously and simultaneously and that the paths associating them with the muscles transmit impulses to and from the muscles in a normal manner. It is a well known fact that when the pyramidal mechanism is disturbed in its function, there is an increased muscle tone as indicated by the exaggerated reflexes. When the cerebellar mechanism is disturbed in function, hypotonia is a prominent symptom. If the cord is completely interrupted above the lumbar enlargement, interrupting all impulses to and from the cerebellum and cerebrum, there is a complete loss of tone of all the muscles enervated below the lesion, and a total loss of all reflexes, the paralysis resulting being a true flaccid paralysis.

Very few spasmodic affections can be traced to the region of the lower motor neurons. This is true whether the lesion be situated in the ventral horns of the grey matter of the spinal cord, or involves the motor fibers of the peripheral nerves. It is not unusual, however, for it to occur as a result of irritation of sensory nerve fibers. Irritation of the nerves of the teeth may start a facial spasm, corneal ulcer may cause a tonic contraction of the orbicularis. Palpebrarium and inflammation of a joint excite spasm of its muscles. These are examples of the so-called reflex spasms.

From the above statement it can be readily seen that spasmodic affections result from a variety of pathological conditions, and for that reason cannot be given a definite association with any particular pathological condition; but there is justification for grouping together in a clinical sense various spasmodic affections whatever be their pathological cause or causes.

As stated above, chorea belongs to the class of spasmodic affections. We note three principal types of chorea—the so-called Sydenham's chorea, Huntington's or hereditary chorea, and senile chorea. In addition, we have chorei-form movements associated with lesions of a mild and gross nature situated in the rubrothalamic region.

It is not my purpose at this time to discuss Huntington's chorea or senile chorea further than to make brief reference to them in passing. Huntington's chorea is a somewhat rare disease, was first described by Huntington in 1872. It is characterized by the appearance in middle life of involuntary purposeless movements in conjunction with a progressive mental deterioration. The symptoms are usually manifest in the fourth decade, but may develop at a later period. Several members of the same family are usually affected, and it may be traced back through several generations. Spasmodic movements are slower and more jerky than those of ordinary chorea and the inco-ordination is greater. In the early stages of the disease, movements may be slight and very limited in their distribution; later they become more violent and may involve practically all of the muscles of the body. The ocular muscles being involved giving a rolling movement of the eyes, face showing grimaces, the tongue contorted, words and syllables badly pronounced, and may be interrupted by inspiratory and expiratory noises. As the disease advances the ability of the patient to control the movements by an act of will or voluntary movement steadily decreases and the movements may cease only during sleep. The unsteadiness of gait and station give evidence of the inco-ordination. Deterioration of the mental faculties is progressive and characteristic. The most constant pathology of hereditary chorea is a diffuse encephalitis. Chronic in character, frequently occurring in the form of a diffuse miliary sclerosis and often associated with a chronic pachymengitis. In other instances the lesion may appear as a primary parenchymatous degeneration of the cortical cells. There is no treatment known at this time which will delay or alter the progress of hereditary chorea. Symptoms may be mitigated by the administration of arsenic and by the use of sedatives such as the bromides.

### SENILE CHOREA.

Senile chorea resembles the other types of chorea in its manifestations. It is a disease of advanced years, usually occurring after the forty-fifth year. It may develop following a fright. It is not ordinarily associated with rheumatism as Sydenham's chorea, nor does it show any hereditary tendencies. It does not shorten life nor does it grossly impair the mental faculties. It does not tend to get well, although it may subside after a year or so. The best known pathology is that of a cortical degeneration similar to the parenchymatous degeneration of hereditary chorea. It should be treated by tonics such as arsenic and iron, by aspirin and sedatives.

Sydenham's chorea is the most common type with which we have to deal, is the most favorable in prognosis, and has a most satisfactory therapy. It is essentially a disease of childhood, the majority of cases occurring between the ages of five and sixteen years. It is characterized by involuntary spasmodic movements and often by muscular weakness and psychical changes. It affects girls more frequently than boys, being in a ratio of about three to one, is occasionally associated with pregnancy, is very rarely seen in males after the twentieth year, but females do not usually have it after this age. Strictly speaking, it is not hereditary but one or both parents may have suffered with chorea or there may be some family history of epilepsy or insanity, or there may be a neurotic taint. A family history of rheumatism, however, is more commonly seen than is a history of neurosis.

Chorea is very closely associated with rheumatism and endocarditis. It may be associated with or be the sequel of an acute attack of rheumatism of a limited or extensive nature. Even though there be no history of a rheumatic attack there is commonly evidence of a rheumatic taint, such for example as the existence or pre-existence of subcutaneous nodules, or erythematous eruptions with slight pyrexia, tonsilitis, or of pains in the joints or limbs with slight effusions in the joints. It is frequently associated with endocarditis, which is another evidence indicating its rheumatic origin.

In all cases of chorea a careful investigation should be made of the tonsils, joints and heart for evidences of a rheumatic infection.

### RELATION TO EMOTION.

While a rheumatic foundation can usually be demonstrated for chorea, the acute attack may be precipitated by an emotional disturbance. Sudden fright, intense grief and the shock of trauma may immediately precede the development of an attack.

### RELATION TO PREGNANCY.

If the young pregnant female be emotionally disturbed, the tendency to chorea is greatly increased. This explains why choera is more commonly present among young girls and women who become pregnant out of wedlock than it is among those who are married. The emotional disturbance of a first pregnancy also predisposes to chorea. If it occurs during the first pregnancy, it may occur during a second or third but rarely does the initial attack occur after the first pregnancy. It is during the third month of pregnancy that the attack usually begins. It occurs rarely following an abortion or after parturition at full term.

RELATION TO HYSTERIA.

Chorei-form movements may occur in association with an hysterical attack, and hysterical manifestations may occur during the course of an attack of chorea. Epidemics of chorea have been reported, but it is not likely that chorea spreads by imitation. Such so-called epidemics are doubtless hysterical attacks with choreiform movements.

SYMPTOMS OF CHOREA.

The choreic movements may develop primarily, but preceding the movements there is usually a characteristic change in the child. It becomes more fretful and irritable, inattentive and apathetic, or there may be manifest symptoms of disturbed function of the extremities such as dragging the leg or a tendency to drop articles from the hand, these symptoms being due to irregularities of contractions of muscles and to muscular weakness. Movements or twitching of muscles usually begin in the face or one hand, but they may begin elsewhere. They may be limited to a few of the muscles, although in severe cases all the muscles of the body seem to be involved.

After the case has developed, the picture is somewhat as follows: The eyebrows are quickly raised and lowered, the eyes are alternately opened and closed, the eyeballs are rotated, the corners of the mouth are raised and then depressed, or the mouth rapidly opens and then closes, the tongue may be quickly protruded and retracted. These movements give a rapidly alternating facial expression indicating delight, surprise, vexation and anger. The movements of the head may be violent in nature, they may be from side to side or backward and forward. The movements of the upper limbs are of great variety and extensive in character. Shoulders shrugged, arms rotated, abducted, constantly moved forward and backward. The forearm and wrist are alternately flexed, extended and rotated, the hand being rapidly changed from pronation to supination. The hand is opened and closed with rapidity and violence. Inco-ordination greatly interferes with all voluntary acts. The movements of the muscles of the trunk cause writhing and twisting movements of the body. Movements of the legs are also of great variety, the feet being inverted and everted, the leg is flexed and extended, rotated, abducted and adducted, and while walking is thrown hither and thither. In all cases of leg involvement, the walking is difficult and in some cases impossible. The inco-ordination of the muscles of articulation and of respiration causes considerable alteration of speech. Words are uttered in a quick jerky manner and sentences may be interrupted by irregular inspiratory movements. Speech disturbances may be so extreme as to amount to an actual aphasia. Muscles of mastication may also be affected so as to greatly interfere with the performance of these acts. The movements, if mild in character, may be inhibited by an act of the will; but in the more severe types a voluntary co-ordinate movement cannot be accurately executed. In many cases the patient is unable to cause relaxation of the choreic spasm, while in others a very obvious muscular weakness impairs function. This weakness may be present in a limb without any movement at all and seems to have no relation to the severity of the movement. An actual paralysis may be present, being monoplegic, hemiplegic or paraplegic in distribution. It may be partial and is rarely complete. There is but little atrophy of muscles, and the paralysis is not permanent.

SENSORY SYMPTOMS.

Spontaneous pains and headache are commonly present in the early stages. Chorea does not cause anesthesia. A peripheral neuritis or hysteria associated with the chorea may give this symptom.

THE REFLEXES.

The deep reflexes in chorea are generally decreased. They may be increased or normal. A peculiar phenomenon sometimes seen in chorea, and often mistaken for an exaggerated knee jerk, is a pseudo-reflex or sustained knee jerk following irritation of the tendon, which irritation causes the choreic spasm. Superficial reflexes are also variable. They may be brisk, diminished or absent. The abdominal reflex may be absent on the affected side in hemi-chorea with an extensor toe phenomenon.

MENTAL CONDITION.

Reference has been made to the inattention and apathy of the child affected with chorea. Mental dullness may be so extreme as to amount to an actual dementia. Delirium and maniacal manifestations may be symptoms. Heart disease is not infrequently associated with chorea; it may be the result of a previous attack of rheumatism. Valvular disease is present in a very high percentage of those cases of chorea in which death results. Pericarditis and endocarditis are frequently present during the course of the disease. Unless there be present arthritis and endocarditis, there is usually no elevation of temperature in chorea. Hyperpyrexia may occur in association with endocarditis and delirium. I have personally seen this present in two fatal cases. In addition to the ordinary variety of Sydenham's chorea, as described above, there occur cases out of the ordinary type. Those cases in which the movements are especially violent and seem to involve all the muscles of the body have been designated as chorea gravis. In these cases the patients are unable to execute any purposeful movements, speech is unintelligible and it is necessary that restraint be used in order to keep patient in bed. These severe types frequently result fatally. Those cases in which the movements are exclusively limited to the limbs of one side are called hemi-chorea. The muscles of the face and trunk are always bi-laterally affected. They are not involved in hemi-chorea. In some cases of chorea the most prominent symptom is the loss of power in a limb. The arm is more frequently involved than the leg. Weakness comes on gradually and there is hypotonia with loss of deep reflexes.

PATHOLOGY.

No very definite. or constant pathology of chorea has been demonstrated. The vascular changes with exudation of hemorrhage around the vessels with embolism and small spots of softening so frequently found are not peculiar to this disease. The most constant morbid condition is an endocarditis. The clinical manifestations would indicate that the lesion is cortical. According to Bury, this is indicated by the frequent excitation of an attack of chorea by fright or by some other form of emotion, by the cessation of the movements during sleep, by the effect on the movements of attention and volition, by the phenomena of hemi-chorea and a hemi-paresis. Spasmodic movements indicate that the motor center of the cortex is implicated. A hypotonia and incoordination also indicate an involvement of the cerebellum. The cause of the lesion is doubtless due to toxemia. Poynton, Holmes and Payne found changes of a vascular and inflammatory nature of the brain and its membranes and also in the nerve tissue itself, consisting of destructive lesions secondary to the vascular changes and of alteration in the morphological character of the nerve cells, such changes being universal throughout the brain. It is the author's belief that they are due to the action of bacterial toxins, a diplococci such as found in rheumatism is found in the meninges and walls of the vessels. The same organisms have also been found in the cerebro-spinal fluid in cases of chorea. The most likely pathology of chorea depends on multiple small scattered lesions throughout the cerebrum and cerebellum. These lesions are probably caused by the action of bacteria of an identical or similar nature to the bacteria responsible for rheumatism and endocarditis, or rather responsible for the arthritis and endocarditis of acute rheumatism. The involuntary movements and slight paresis commonly present are the result of the action of the bacterial toxin upon the cortical motor cells, the hypotonia and inco-ordination due to changes in the cerebellum, paralysis in association with slight anesthesia of the distal portions of an extremity is the result of action of the toxin on the spinal cord or a neuritis of a peripheral nerve.

A diagnosis of chorea is not difficult. The movements of chorea are always purposeless. Chorei-form movements of hysteria are purposeful in character. In some cases in which the movements are light and the mental symptoms rather severe, or if the mental symptoms be well developed and the motor symptoms of chorea slight, there is danger that the chorea will be overlooked and the case regarded as one of katatonia or manic depressive insanity.

This latter type of insanity usually attacks those who are older. Paralysis may precede the choreic movements for some time, even several weeks. Paralysis in one arm with gradual onset in a child is a strong suggestion of chorea, and in all cases of paralysis, unless the origin of same is definitely known, a child should be watched very carefully for spontaneous movements, otherwise a chorea may be overlooked.

### PROGNOSIS.

A vast majority of cases of chorea recover, recovery usually taking place within two months, even in severe cases. But, even though recovery take place, there is a tendency to recurrence. The prognosis is not favorable in chorea of pregnancy, or in those cases where there is much psychical disturbance. A patient may die from exhaustion, but death is usually the result of endocarditis, pericarditis, embolism and hyperpyrexia.

### TREATMENT.

The prophylactic treatment of chorea is important. Many children are so emotionally unstable that they have a natural chorea. Their inhibitory powers are very poorly developed, and emotion and desire is quickly transferred into muscular activity. Children who are predisposed to chorea are active, irritable, restless, fretful under restraint, much disposed to carry out forbidden acts. They are generally tall, with narrow, long chests, osseous and muscular systems, are poorly developed; they have adenoids and large tonsils. Such children should spend much time out of doors, should be fed a simple but nutritious diet, sleep long hours, and should not be rushed through school. Adenoids and large tonsils should be removed, the tonsils being thoroughly enucleated.

After an attack of chorea develops the medical treatment is important. Much can be done by an intelligent management of the child. All school work should be at once discontinued, playing and violent exercise of all sorts should be forbidden. It is not necessary that the child should be put to bed, but it should spend a part of the day lying down. A simple, nutritious diet without meat should be given. If able, the child should be out in the open a considerable part of the day, and the room in which it sleeps should be well ventilated. If the movements are inclined to be at all violent or severe, putting the patient in a wet pack at 70 degrees and keeping him there for a period of two hours will be found to be very helpful in controlling the movements and as a general tonic. If the patient is much debilitated, iron and arsenic will be found helpful as a tonic. Arsenic has long been used for chorea or in the treatment of chorea. I do not believe it has any specific action in chorea, but consider it to be a good tonic. By the mouth, I give Fowler's solution, to the physiological effect, or it can be given by a hypodermic in the form of cacodylate of soda, an injection being made once or twice or more times per week. Iron may be given in the form of Blaud mass, solution of albuminate of iron, etc. Attention should be given to the condition of the bowels, and they should be kept free. If cardiac symptoms develop, they should be treated symptomatically. The delirium can best be controlled by a wet pack or by sedatives, if found necessary to give them.

The drugs thus far recommended are used principally for the purpose of controlling symptoms. There is one drug which I consider to be practically specific in the treatment of chorea, and that is aspirin. It should be given from the onset and in doses ranging from twenty to sixty grains per day according to age. Naturally, more can be given with safety if the patient be in the hospital and under daily observation of a physician. Large doses are preferable and are practically safe. I have rarely seen a case of chorea extend beyond a period of three weeks when treated by aspirin. Dr. Forquahr Buzzard of the National Hospital, Queen's Square, London, says that before using aspirin in the treatment of chorea, when a patient came to him in the out-patient department, he felt very sure that later it would be necessary for it to enter the hospital and be put to bed; but since he has used aspirin he has never had to bring a patient into the hospital who first came to him in the out-patient department.

It has been my experience that patients with chorea begin improving in a very few days and do not get worse after they begin taking aspirin. The arsenic and other remedies have not given like results. The rheumatism phylocagens I have not tried. Theoretically they should be of some value. Arsenic should not be given to a pregnant choreic, in large doses, for fear of injuring the unborn child. For the same reason sedatives should be given with extreme care. Strontium bromide is the safest. Most cases in which the movements are prolonged the re-education method of Meige and Findel, the inhibition theory of Openheim and respiratory exercises of Pitres can be resorted to. Any method of treatment which will increase the attentive control and develop the power of inhibition will be found useful.

